# Genomic Medicine in the Developing World: Cancer Spectrum, Cumulative Risk and Survival Outcomes for Lynch Syndrome Variant Heterozygotes with Germline Pathogenic Variants in the *MLH1* and *MSH2* Genes

**DOI:** 10.3390/biomedicines12122906

**Published:** 2024-12-20

**Authors:** Lutricia Ndou, Ramadhani Chambuso, Ursula Algar, Adam Boutall, Paul Goldberg, Raj Ramesar

**Affiliations:** 1 UCT/MRC Genomic and Precision Medicine Research Unit, Division of Human Genetics, Department of Pathology, Institute of Infectious Disease and Molecular Medicine, University of Cape Town, and Affiliated Hospitals, Cape Town 7704, South Africa; ndxlut003@myuct.ac.za (L.N.); ramadhani.chambuso@uct.ac.za (R.C.); 2 The Colorectal Unit, Department of Surgery, Groote Schuur Hospital, The University of Cape Town, Cape Town 7925, South Africa

**Keywords:** Lynch syndrome registry, colorectal cancer, extracolonic cancer, cumulative risk, genetic variants, crude survival, South African founder variant, pathogenic variants

## Abstract

**Background**: Although genetic testing has improved our ability to diagnose Lynch syndrome (LS), there is still limited information on the extent of variations in the clinical and genetic landscape among LS variant heterozygotes (LSVH) in Africa. We sought to investigate the cancer spectrum, cumulative risk, and survival outcomes of LSVH with pathogenic/likely pathogenic variants (P/LPVs) in the *MLH1* and *MSH2* genes using a LS registry in South Africa over the last 30 years. **Methods**: A retrospective study was conducted to retrieve demographic, clinical, and genetic data of all LSVH with P/LPVs in the *MLH1* and *MSH2* genes from our LS registry. Genetic data were analyzed according to cancer spectrum, cumulative risk, and crude survival. We used the Chi-squared and *t*-test to assess differences between groups, and Kaplan–Meier survival analyses were used to analyze the cumulative risk and crude survival outcomes. A *p*-value < 0.05 at a 95% confidence interval was considered statistically significant. **Results**: We analyzed a total of 577 LSVH from 109 families. About 450 (78%) and 127 (22%) LSVH harbored a disease-causing mutation in *MLH1* and *MSH2*, respectively. A South African founder PV (*MLH1*:c.1528C>T) accounted for 74% (n = 426) of all LSVH. CRC was the most common diagnosed cancer in both *MLH1* and *MSH2* LSVH. *MLH1* LSVH had a younger age at cancer diagnosis than *MSH2* LSVH (43 vs. 47 years, respectively, *p* = 0.015). Extracolonic cancers were predominantly higher in female LSVH (n = 33, 35%) than in male LSVH (n = 8, 7%) with the *MLH1*:c.1528C>T founder PV. The cumulative risk of any cancer and CRC at any age was higher in *MLH1* LSVH than in *MSH2* LSVH (*p* = 0.020 and *p* = 0.036, respectively). LSVH with the *MLH1*:c.1528C>T PV had a better 10-year overall survival after the first cancer diagnosis, particularly for CRC. **Conclusions**: LSVH with P/LPVs in the *MLH1* and *MSH2* genes exhibited significant gene- and sex-specific differences in cancer spectrum, cumulative risk and survival outcomes. Cancer risk and survival estimates described in this study can be used to guide surveillance and genetic counselling for LSVH in our population.

## 1. Introduction

Cancer is a growing public health problem, and approximately 10% of all cancers can be attributed to hereditary cancer syndromes which are led by Lynch syndrome (LS) [1,2,3,4,5]. LS is the most common hereditary cancer-predisposing syndrome which accounts for 3–5% of all colorectal or endometrial cancers, and 10–15% of tumors with DNA mismatch repair (MMR) deficiency [6]. LS is caused by a mono-allelic inherited pathogenic variant (PV) in one of the four MMR genes: *MLH1*, *MSH2*, *MSH6*, or *PMS2*, or by a deletion of the 3′ end of the *EpCAM* gene [7,8,9]. Among the four MMR genes mentioned above, mono-allelic inherited PVs in *MLH1* and *MSH2* are the most common and account for approximately 70–90% of all LS cases, while 7–10% are due to PVs in the *MSH6* gene and less than 5% are due PVs in the *PMS2* gene [10,11]. In addition, approximately 1% of the LS cases are due to deletions in the *EpCAM* gene, which silences the downstream *MSH2* gene [12,13]. The lifetime risk of developing cancer for LS variant heterozygotes (LSVH) ranges from 30 to 80% and at a relatively younger age than the general population [6].

LS is characterized by high variability in cancer manifestation, even in LSVH with the same germline PV [14,15]. The current understanding of cancer manifestation variability in LSVH is predominantly based on Caucasian populations (rather than African populations) [13,14,15,16,17,18,19]. Different populations may have distinct genetic backgrounds influencing the manifestation of germline PV in the MMR gene, necessitating the need for population-specific cancer risk and survival outcomes analysis for precise clinical surveillance in LSVH [20,21]. However, this analysis is currently lacking in African populations, underscoring the need for genetic characterization of LSVH in Africa to enhance clinical characterization and improve management and surveillance of high-risk patients and their families [12,16,22,23,24,25].

A publicly available LS database, the Prospective Lynch Syndrome Database (PLSD), which is mainly comprised of LSVH from European, North American, South American, and Asian countries, has consistently shown that the cancer risks associated with each MMR gene differ despite similar surveillance guidelines [13,14,15,16]. The median age of cancer onset in LSVH has been reported to range from 48 to 64 years, depending on the mutated MMR gene [19,24,26]. LSVH with germline PVs in the *MLH1* and *MSH2* genes are known to have a higher risk of developing colorectal, endometrial, and ovarian cancers [13,19,24]. However, LSVH with germline PVs in the *MSH2* gene also have an increased risk for other solid cancers such as brain, upper gastrointestinal, urinary tract, and prostate [13,19,24]. Conversely, LSVH with germline PVs in the *MSH6* gene have an increased risk of endometrial cancer and a slightly higher risk of colorectal cancer (CRC), whereas LSVH with germline PVs in the *PMS2* gene have a lower risk of developing any cancer [16]. It has been shown that male LSVH have a higher lifetime risk and early age of cancer manifestation compared to female LSVH [17,18,19]. In addition, some cancers such as bladder, gastric, and urothelial have been reported to be much higher in males than in females [17].

Hereditary cancer registries, particularly LS registries, have been used to study cancer risk and evaluate clinical surveillance outcomes for CRC, ultimately improving the clinical management of LS families and reducing mortalities associated with LS cancers [27,28]. In South Africa, we have a large LS registry in the Division of Human Genetics at the University of Cape Town (DHG-UCT), which is resource used for the multidisciplinary team managing patients/families with LS [29,30,31,32,33].

In this study, we investigated LSVH with germline pathogenic/likely pathogenic variants (P/LPVs) in the *MLH1* and *MSH2* genes in our LS registry and evaluated the spectrum of solid cancers, cumulative risk, and survival outcomes.

## 2. Materials and Methods

### 2.1. LS Registry at the DHG-UCT

**Figure 1:**
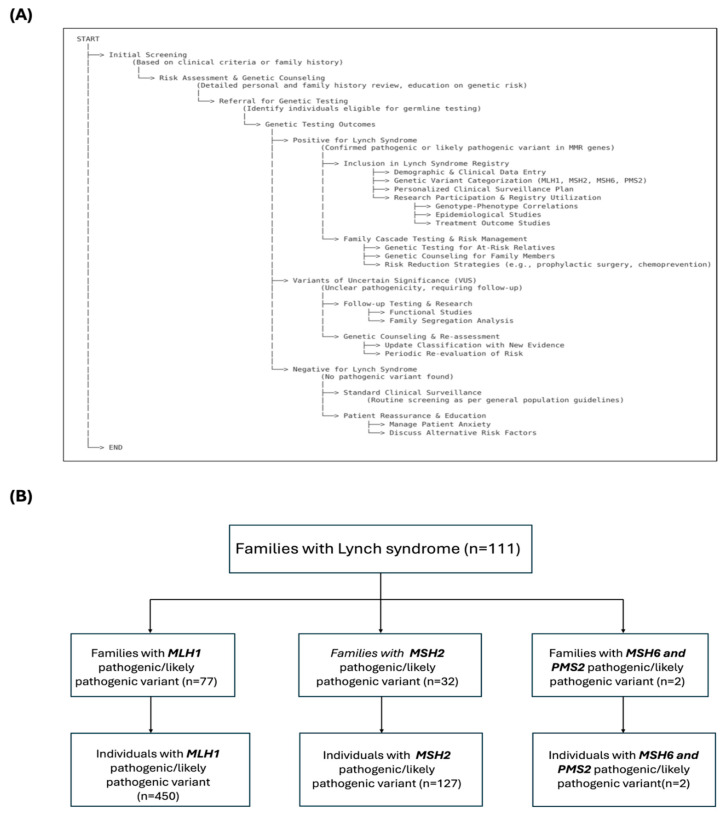
A comprehensive description of our LS registry and patient recruitment. (**A**) The flowchart outlines a step-by-step structured process for managing and categorizing individuals suspected or confirmed to have LS. It begins with the “Initial Screening” based on clinical criteria or family history (Bethesda and Amsterdam criteria I/II for LS), followed by Risk Assessment & Genetic Counselling. Individuals meeting eligibility criteria are referred for *Genetic Testing*, leading to three distinct outcomes: Positive for LS, Variants of Uncertain Significance (VUS), or Negative for LS. Each outcome has specific downstream pathways for inclusion in the LS registry, follow-up testing, or standard clinical surveillance, respectively. The flowchart also details strategies for Family Cascade Testing, comprehensive management, and research integration. (**B**) A flow diagram showing the number of families and individuals who were genetically diagnosed with LS in our registry, categorized by the specific MMR gene with germline P/LPVs. Note: While families and individuals with *MSH6* and *PMS2* P/LPVs are shown here for completeness, they were excluded from further analyses due to small number of cases.

As a part of a large ongoing molecular genetic study aimed at identifying individuals at high risk of developing LS-associated cancers for inclusion in the clinical surveillance program at the DHG-UCT, a total of 111 families with 579 individuals have been genetically diagnosed with LS (Figure 1). The first patients (also known as probands) to be diagnosed with LS in the family were identified using the Bethesda and Amsterdam criteria I/II testing guidelines for LS at Groote Schuur Hospital (GSH) and referred for germline genetic testing to the DHG-UCT or the National Health Laboratory Services at UCT (NHLS-UCT) [34,35,36,37]. This initiative started in 1997, the demographic, clinical, pathological, and genetic information of these individuals is stored in our in-house LS registry at the DHG-UCT (Figure 1).

### 2.2. Genetic Laboratory Analysis

Genomic DNA (gDNA) of individuals with LS represented in our registry was extracted from peripheral blood samples using either the manual salting-out or automated magnetic bead-based (Maxwell@ 16, Promega, Madison, WI, USA) methods [38,39]. From 1997 to 2016, genetic testing for germline variants in the MMR genes was conducted using polymerase chain reaction amplification of a specific MMR gene in the DHG-UCT. Since 2016, the testing was transitioned to the NHLS-UCT, where direct sequencing for the five (5) most common Mixed ancestry variants (NM_000249.4 (*MLH1*):c.1528C>T, NM_000251.3(*MSH2*):c.387_388deITC, NM_000251.3(*MSH2*):c.1221_1222deICT, NM_000251.3(*MSH2*):c.1340_1341insGG, and NM_000251.3(*MSH2*)c.1046C>G) was performed using the automatic DNA sequencer (Model ABI-3100, Applied Biosystems, Foster City, CA, USA) and/or Snapshot analysis for individuals with a definite family history of LS or with Immunohistochemistry (IHC) for MMR proteins showing loss of nuclear tumor staining (MMR protein deficiency). Additionally, next-generation sequencing (NGS) using a 14-gene panel for hereditary CRC (*APC*, *BMPR1A*, *EPCAM*, *MLH1*, *MSH2*, *MSH6*, *MUTYH*, *PMS2*, *POLD1*, *POLE*, *PTEN*, *SMAD4*, *STK11*, and *TP53*) was used for cancer-affected patients without family history or with IHC for MMR proteins showing intact/retained nuclear staining (MMR protein proficiency) (Applied Biosystems, ThermoFisher Scientific, MA, USA). For patients with no germline PV identified in the MMR genes, their samples were investigated for large genomic rearrangements involving *MLH1*, *MSH2*, and *EPCAM* using the multiplex ligation probe amplification (MLPA) SALSA P003 kit according to the manufacturer’s specifications (MRC-Holland, Amsterdam, The Netherlands).

### 2.3. MMR Variant Nomenclature and Classification

The germline MMR variants in our LS registry were described using the Human Genome Variation Society (HGVS) nomenclature guidelines and classified according to the International Society of Gastrointestinal Hereditary Tumors (InSIGHT) [40,41]. The following reference sequences were used to describe cDNA variants in MMR genes, NM_000249.4 (*MLH1*) for *MLH1* gene variants, NM_000251.3 (*MSH2*) for *MSH2* gene variants, NM_000179.3 (*MSH6*) for *MSH6* gene variants, and NM_000535.7 (*PMS2*) for *PMS2* gene variants (https://www.ncbi.nlm.nih.gov/clinvar/, accessed on 8 October 2024). The references used to describe protein changes were as follows: NP_000240.1 for MLH1, NP_000242.1 for MSH2, NP_000170.1 for MSH6, and NP_000526.2 for PMS2 protein changes (https://www.ncbi.nlm.nih.gov/clinvar/, accessed on 8 October 2024).

### 2.4. Study Population

In this study, we only included individuals with germline P/LPVs in the *MLH1* and *MSH2* genes, due to a small number of individuals with variants in the *MSH6* and *PMS2* genes in our LS registry (Figure 1B). We categorized LSVH into three groups to perform our analyses:

(i) Group 1A (N = 450 LSVH) included all LSVH with a germline P/LPV in the *MLH1* gene with any variant (obligate LSVH with or without cancer who were under follow-up for their disease).
(ii) Group 1B (A subgroup of Group 1A but with only *MLH1*:c.1528C>T PV, N = 426 LSVH) included only LSVH with a germline South African founder PV *MLH1*:c.1528C>T (obligate LSVH with or without cancer who were under follow-up for their disease).
(iii) Group 2 (N = 120) included all individuals with a germline P/LPV in the *MSH2* gene (obligate carriers with or without cancer who were under follow-up for their disease).

We retrieved the data from our LS registry, which included personal and family history, cancer diagnosis, age at cancer diagnosis, date of birth, date of death, pathology data, and MMR germline genetic testing results. Pathology reports obtained from the NHLS were also used to confirm the pathology information recorded in the LS registry. The demographic, clinical, and pathological data (including death status obtained from relatives) of individuals included in this study were last reviewed in May 2024.

### 2.5. Statistical Analysis

Statistical analyses were performed using R version 4.3.3 software (https://www.r-project.org/, accessed on 2 April 2024). The Chi-squared test and Fisher exact test were used to compare the frequencies of demographic and clinicopathological characteristics between Group A1 and Group 2. In addition, a student *t*-test was used to determine the difference in mean between the two groups, while the Mann–Whitney U test was only used in the comparison of LSVH diagnosed with cancer within Group 2 due to a small sample size. Group 1B was excluded from the comparison analysis to avoid redundancy, as individuals in this group are all included within Group 1A. The cumulative risk of first cancer was calculated using the Kaplan–Meier method, and we compared the risk curves using a log-rank test. Overall survival (defined as the time from diagnosis date until the date of death from any cause) was estimated using the Kaplan–Meier method, with the analysis limited to cases diagnosed before the age of 65 years to minimize the influence of competing causes of death [42,43]. The *p*-value < 0.05 with a confidence interval of 95% was considered statistically significant.

## 3. Results

### 3.1. Demographics and Clinical Features of the Study Cohort

We analyzed a total sample size of 577 LSVH from 109 families in our LS registry consisting of Group 1A (n = 450), and Group 2 (n = 127). Group 2 had a higher proportion of female LSVH (66.7%) than male LSVH (33.3%), while Group 1A showed an approximately equal sex composition. A statistically significant difference in sex composition was observed between Group 1A and Group 2 (*p* = 0.005, Table 1). The majority of LSVH cases in our study cohort were of mixed ancestry for about 97.5% (n = 439) in Group 1A, and 90% (n = 108) in Group 2. A total of 279 LSVH were diagnosed with cancer (either colonic or extracolonic cancers), in Group 1A, n = 222 and in Group 2, n = 57. CRC was the most frequently diagnosed first cancer than all extracolonic cancers in both groups, with no statistically significant difference between Group 1A and Group 2 (*p* = 0.654). Similarly, the frequency of CRC (79.9%, n = 163) was higher than that of extracolonic cancers (20.1%, n = 41) among LSVH in Group 1B (a subset of Group 1A). Additionally, the frequency of CRC was still the highest across the entire population in both sexes, with no statistically significant difference between Group 1A and Group 2 (Table 1).

### 3.2. Genetic Characterization of LSVH in Our LS Registry

**Figure 2:**
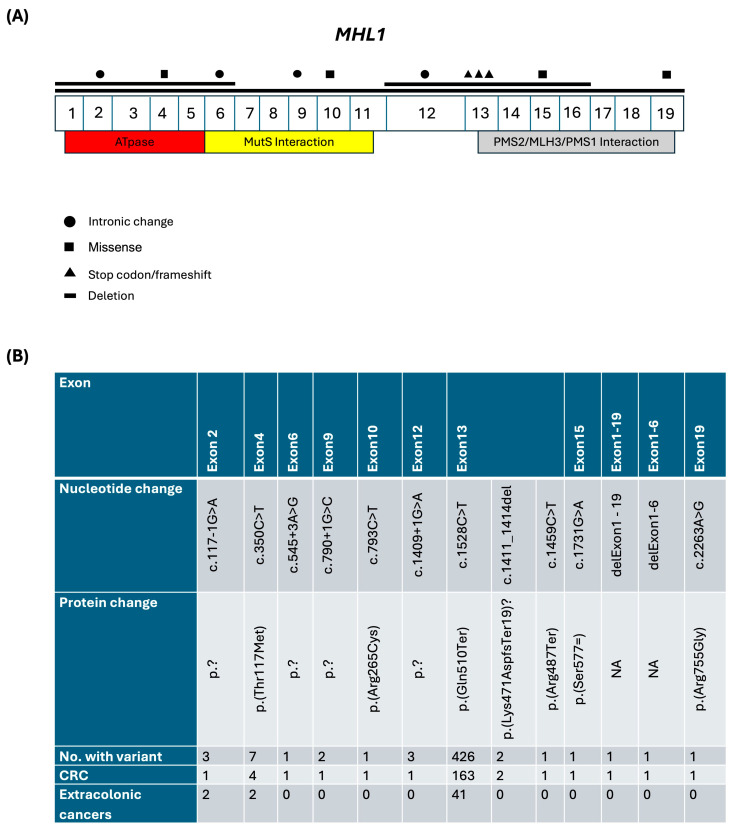
Distribution and clinical characteristics of P/LPVs in the *MLH1* gene. (**A**) The exon-intron structure of the *MLH1* gene, highlighting functional domains: ATPase domain (exons 1–5, red), MutS interaction domain (exons 6–11, yellow), and PMS2/MLH3/PMS1 interaction domain (exons 13–19, gray). Symbols represent different types of variants: intronic changes (●), missense mutations (■), stop codon/frameshift mutations (▲), and deletions (—). (**B**) A description table provides a detailed summary of nucleotide and protein changes for each P/LPV, their corresponding exons, and the number of individuals carrying these PLPVs. Exon 13 shows the highest variant frequency, with 426 LSVH with the PV c.1528C>T (p.Gln510Ter). The associated cancer phenotypes indicate a predominance of CRC among LSVH, while extracolonic cancer incidence remains low.

Understanding the distribution and clinical implications of P/LPVs in the *MLH1* and *MSH2* genes is essential for guiding genetic counselling, targeted testing, and personalized cancer surveillance in LSVH. In our LS registry, there were 35 unique P/LPVs in the *MLH1* and *MSH2* genes. The majority of LSVH (78.0%, n = 450) harbored a P/LPV in *MLH1*. A total of 13 unique P/LPVs were identified in *MLH1*: 3 (23.1%) stop codon/frameshift variants (in exon 13), 4 (30.8%) missense variants (in exons 4, 10, 15, and 19), 4 (30.8%) intronic change (in exons 2, 6, 9 and 12), and 3 (23.1%) large deletions (from exons 1–6, 12–16, and 1–19), Figure 2A. Notably, the most common variant was c.1528C>T with a protein change *p*. (Gln510Ter) in exon 13, with 426 occurrences, primarily associated with CRC (n = 163) and a range of extracolonic cancers (n = 41), Figure 2B.

**Figure 3:**
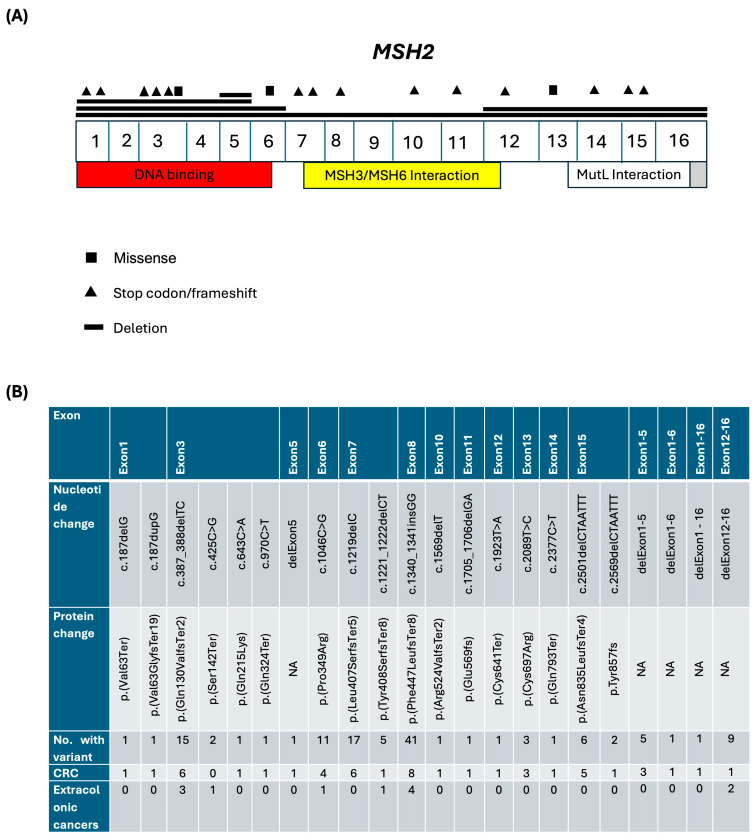
Distribution and clinical characteristics of P/LPVs in the *MSH2* gene. (**A**) A panel displays the exon–intron structure of the *MSH2* gene, illustrating its functional domains: DNA binding domain (exons 1–6, red), MSH3/MSH6 interaction domain (exons 7–12, yellow), and MutL interaction domain (exons 13–16, gray). Different variant types are represented by specific symbols: missense mutations (■), stop codon/frameshift mutations (▲), and deletions (—). (**B**). A table details the nucleotide and protein changes for each PV, the specific exons affected, and the number of LSVH. Exon 7 shows the highest variant frequency, with 17 LSVH with the PV c.1219delC (p.Leu407SerfsTer5). The associated cancer phenotypes indicate a predominance of CRC among LSVH, while extracolonic cancer incidence remains low.

A total of 22 unique PV/LPVs were identified in *MSH2*: 14 (63.6%) stop codon/frameshift variants (in exons 1, 3, 7, 8, 10, 11, 12, 14, and 15), 3 (13.6%) missense variants (in exons 3, 6, and 13), and 5 (22.7%) large deletions (from exon 5, 1–5, 1–6, 12–16, and 1–16), (Figure 3A). In addition, the c. 1340_1341insG PV in exon 8 of the *MSH2* gene was the most frequent PV, accounting for 32.3% (41/127) of all *MSH2*-associated LS cases. Also, CRC (n = 8) was the most frequently diagnosed cancer associated with this c. 1340_1341insG common PV in the *MSH2* gene (Figure 3B).

### 3.3. Age at Colonic and Extracolonic Cancer Diagnosis in LSVH

We analyzed the pattern of age at cancer diagnosis among LSVH which may influence clinical surveillance strategies and risk management. Analysis of the age at any cancer diagnosis reveals a significant gene disparity, with Group 1A showing an earlier mean age at cancer diagnosis compared to Group 2 (43.2 vs. 47.3 years; *p* = 0.015, Table 2). In addition, when further stratified by sex, a significant difference in age at any cancer diagnosis was observed between males in Group 1A and those in Group 2 (41.8 vs. 47.5 years, *p* = 0.017, Table 2). For first CRC, the age at diagnosis was not significantly different between groups (*p* = 0.078); however, for male LSVH, there was a statistically significant difference in the age at CRC diagnosis between Group 1A and Group 2 (41.6 vs. 46.9 years, respectively, *p* = 0.043, Table 2). The distribution of age at any cancer diagnosis in Group 1A and Group 2 stratified by sex is further illustrated in Supplementary Figure S1, while Table S1 provides a summary of the mean age at diagnosis for the six major cancers observed in our study cohort.

### 3.4. Cancer Spectrum and Frequencies in LSVH by Gene and Sex

We evaluated the distribution of cancers and gene-specific variability among LSVH between Group 1A and Group 2 to elucidate the necessity for personalized surveillance and management strategies based on the mutated MMR gene. CRC was the most frequent cancer diagnosed in both Group 1A (n = 176/222; 79.3%) and Group 2 (n = 43/57; 75.4%), accounting for nearly 75% of all cancer cases. Interestingly, breast cancer (BC) was relatively more common in Group 1A (n = 8/222; 3.6%) than in Group 2 (n = 1/57; 1.8%). Moreover, endometrial cancer was identified in 6.8% (n = 15/222) of LSVH in Group 1A compared to 3.5% (n = 2/57) of LSVH in Group 2. Small bowel cancer was the second most common cancer in Group 2 (4/57; 5.3%). Cancers such as bladder, brain, liver, ovary, pancreas, kidney, prostate, and thyroid were observed only in Group 1A, while head and neck, esophagus, spleen, and lung cancers were only observed in Group 2 (Table 3). The proportion of cancers and their incidences for Group 1B is also shown in Table 3.

**Figure 4:**
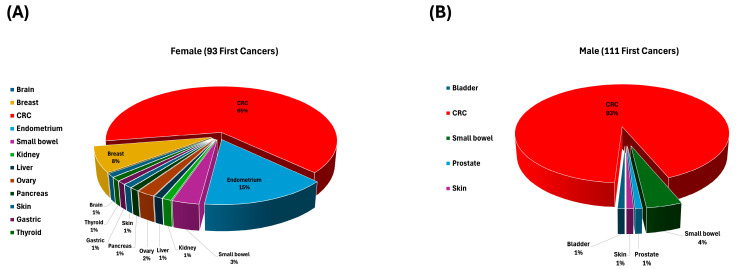
Pie charts showing the distribution of first cancers in LSVH according to sex. (**A**) The spectrum of first cancers in female LSVH (n = 93), where CRC was the predominant first cancer type, accounting for 65% of all first cancers. (**B**) The distribution in male LSVH (n = 111) with a dominance of CRC (93%) as the first cancer diagnosed, with limited occurrence of other cancers.

In addition, as *MLH1*:c.1528C>T founder PV was the most common variant in our cohort, we further stratified this group by sex to highlight any significant differences in first cancer development. A total of 93 female and 111 male LSVH were diagnosed with cancers. In female LSVH, CRC comprised 65% of all first cancer cases observed, while extracolonic cancers comprised about 35% of all first cancer cases, with endometrial cancer being the most frequently diagnosed cancer by 15%, followed by breast (8%) (Figure 4A). In male LSVH, CRC comprised 93% of all diagnosed first cancers, with limited occurrence of other cancers (Figure 4B).

### 3.5. Comparison of Age at CRC Diagnosis Between Prevalent and Incident Cancer Cases

**Figure 5:**
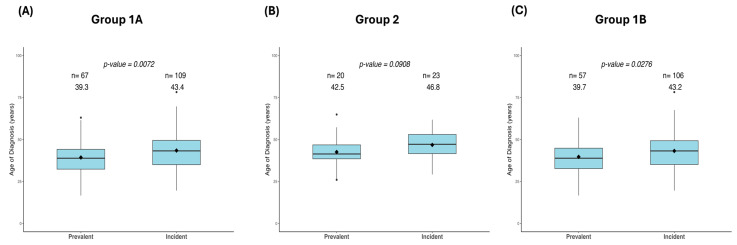
Box plots and whiskers showing age at CRC diagnosis in prevalent and incident cases stratified by mutated gene status. (**A**) In Group 1A, the prevalent cases were diagnosed with cancer at a significantly younger age compared to the incident cases. (**B**) In Group 2, there was no significant difference in the age at cancer diagnosis between the prevalent and incident cases. (**C**) In Group 1B, the prevalent cases were diagnosed with cancer significantly at a younger age compared to incident cases.

We compared whether the age at CRC diagnosis in cases detected at first colonoscopy (prevalent cases) differs from cases detected under regular colonoscopy surveillance (incident cases). A statistically significant difference in the age at cancer diagnosis was observed between prevalent and incident cases in Group 1A, with prevalent cases being detected at a younger age than incident cases (mean age: 39 vs. 43 years, respectively, *p* = 0.007, *t*-test, Figure 5A). However, no significant difference in age at CRC diagnosis was found between the prevalent and incident cases in Group 2, although the mean age at diagnosis for prevalent cases (43 years) was lower compared to that of incident cases (47 years) (*p* = 0.091, Mann–Whitney U test, Figure 5B). In addition, a significant difference was observed between prevalent and incident cases in Group 1B (mean age: 40 vs. 43 years, respectively, *p* = 0.028, *t*-test, Figure 5C).

### 3.6. Gene and Sex-Specific Cumulative Risk of Cancer in LSVH

**Figure 6:**
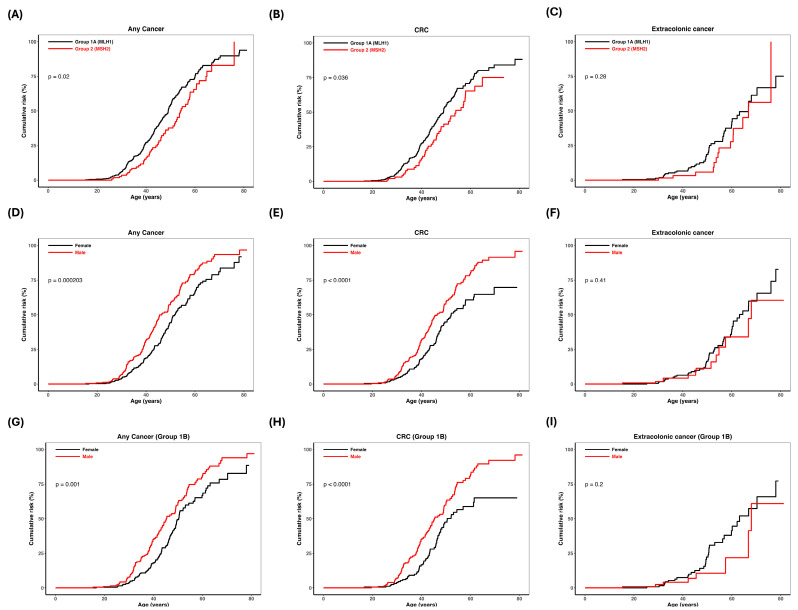
Cumulative risk of LS-associated cancers by gene and sex. (**A**–**C**) The cumulative risk curves comparing *MLH1* (black) and *MSH2* (red) LSVH. (**D**–**F**) Cumulative risk of LS-associated cancers by sex (male = red, female = black). (**G**–**I**) Results in Group 1B, showing similar sex-specific trends.

To better understand the influence of mutated MMR gene (*MLH1* and *MSH2*), founder variant (*MLH1*:c.1528C>T), and sex on cancer risk in LSVH, we calculated the cumulative risk of any cancer (i.e., both colonic and extracolonic cancers combined), colonic, and extracolonic cancers separately using Kaplan–Meier analysis across three distinct groups. This analysis was essential for identifying specific groups that may require tailored surveillance strategies in our population. The risk of any cancer and CRC was significantly higher in Group 1A compared to Group 2 (log-rank test *p* = 0.020 and *p* = 0.036, Figure 6A and Figure 6B, respectively), whereas extracolonic cancer risk did not differ significantly (log-rank test *p* = 0.280, Figure 6C). The cumulative risk of any cancer was 54.2–88.4% and 37.7–82.9% at the age of 50–70 years for Group 1A and Group 2, respectively (Table 4). The cumulative risk of CRC was 54.5– 84.1% and 41.3–75.0% at the age of 50–70 years for Group 1A and Group 2, respectively (Table 4). When stratified by sex, male LSVH had a markedly increased risk of any cancer and CRC compared to female LSVH (*p* < 0.0001 and *p* < 0.0001, Figure 6D and Figure 6E, respectively), while the risk of extracolonic cancer did not differ significantly (log-rank test *p* = 0.410, Figure 6F). The cumulative risk of any cancer in male LSVH was 58.0–93.5% at the age of 50–70 years compared to 44.2–81.0% in female LSVH (Table 4). The cumulative risk of CRC in male LSVH was 58.6–91.5% at the age of 50–70 years compared to 44.7–69.7% in female LSVH (Table 4). We observed a similar trend in risk of any cancer and CRC in LSVH with the *MLH1*:c.1528C>T PV, with male LSVH showing a significantly higher risk than female LSVH (log-rank test *p* = 0.010 and *p* < 0.001, respectively), with cumulative risks of 60.8–94.0% and 49.3–78.5% for any LS cancer, and 60.9–92.2% and 47.5–65.1% for CRC at the age of 50–70 years, respectively (Figure 6G,H and Table 4).

### 3.7. Crude Survival by Sex and Cancer Type in LSVH with MLH1:c.1528C>T PV

We determined the 5-year and 10-year crude survival after the first cancer diagnosis diagnosed before the age of 65 years by sex and cancer type in Group 1B only (due to a lack of survival information i.e., death status for other LSVH in this cohort). The results indicated that 77.6% (95% CI 68.8–87.5%) of female LSVH survived 10 years or more after their first cancer diagnosis, whereas only 62.1% (95% CI 52.9–74.1%) of male LSVH achieved this survival duration (Table 5). The 10-year survival following CRC diagnosis was 70.9% (95% CI 63.5–79.2%), while the survival after the first extracolonic cancer diagnosis was 63.6% (95% CI 47.4–85.3%) (Table 5). However, the Kaplan–Meier survival curves showed no statistically significant difference in survival between female LSVH and male LSVH (log-rank *p* = 0.170) (Supplementary Figure S2A). Also, there was no statistically significant difference in survival between LSVH with CRC and those with extracolonic cancer (log-rank *p* = 0.480) (Supplementary Figure S2B). Gynecological cancers had excellent 5-year and 10-year crude survival compared to CRC; however, the difference was not statistically significant (log-rank *p* = 0.610) (Supplementary Figure S2C). As expected, patients with cancers diagnosed below the age of 40 years showed better survival compared to those diagnosed above the age of 40 years (log-rank *p* = 0.050, (Table 5 and Supplementary Figure S2D).

## 4. Discussion

This study reveals novel insights into LS genetics with cancer spectrum, risk, and survival outcomes from an African population with germline P/LPVs in two MMR genes: *MLH1* (Group 1A) and *MSH2* (Group 2) in South Africa. A high frequency of the *MLH1*:c.1528C>T founder germline PV (Group 1B) in exon 13 was observed, accounting for more than 70% of all LSVH, suggesting a significant founder effect in the study population. CRC was the most frequently diagnosed cancer than any other cancer in the study cohort. Group 1A showed a higher cumulative risk of cancer and had a younger age at cancer diagnosis than Group 2, highlighting gene-specific differences in cancer risk. Additionally, sex-specific differences were observed, with male LSVH showing a markedly higher cumulative risk of cancer and a younger age at cancer diagnosis compared to female LSVH. Notably, LSVH with CRC detected at first colonoscopy (prevalent cases) were diagnosed at a younger age compared to LSVH detected under regular colonoscopy (incident cases) as part of our surveillance. Female LSVH in Group 1B showed a better ten-year crude survival after the first cancer diagnosis compared to male LSVH in the same group. Our study contributes to the global LS knowledge by offering critical, population-specific findings for genetic counselling, risk prediction, and tailored interventions.

The genetic characterization of LSVH in our LS registry highlights distinctive mutation patterns within the *MLH1* and *MSH2* genes. This detailed variant mapping, illustrated in Figure 2 and Figure 3, provides valuable insight into the mutation landscape and its clinical impact, revealing a predominance of stop codon/frameshift variants in *MSH2* gene, whereas *MLH1* variants include a notable mix of intronic and large deletion variants, each contributing differently to cancer risk. The frequency of P/LPVs in the MMR genes in individuals from European populations has been reported as follows: ~15–40% in *MLH1*, ~20–40% in *MSH2*, ~12–35% in *MSH6,* ~5–25% in *PMS2,* and ~10% in *EPCAM* [6,44,45,46,47,48]. The proportion of *MLH1* P/LPVs in our LS registry (78.0%) significantly exceeded the upper limits of the ranges reported from the abovementioned other populations (~15–40%). This discrepancy could suggest a notably higher frequency of *MLH1* P/LPVs in our study population compared to other populations, which could be due to differences in ethnicities, or the sample sizes used. In addition, it could be due a founder effect of *MLH1*:c.1528C>T founder PV, which contributed to more than half of all LS cases in our LS registry. A founder variant is a genetic variant that originates from a small group of ancestors and is subsequently transmitted through generations within a specific population. This type of variant is known to contribute to a substantial proportion of LS cases in various populations due to several factors: certain founder variants are prevalent in specific ethnic or geographic groups; populations that experience genetic bottlenecks—wherein only a limited number of individuals contribute to subsequent generations—are more likely to exhibit a higher frequency of founder variants; and founder variants often possess distinct genetic signatures that facilitate their detection through targeted genetic testing [49,50,51,52].

The founder PV in our study population contributed to 94.7% of all *MLH1*-associated cases and 73.8% of all LSVH in our registry. This *MLH1* founder PV results in a truncated MMR protein and has been traced to originate in individuals of mixed ancestry on the West Coast of South Africa, hence a higher proportion of mixed ancestry individuals in our LS registry [49,53]. Our affiliated hospitals are situated in this region where this founder germline PV originates, thereby explaining the high proportion of individuals with this PV in our LS registry [31,54]. Our findings highlight a significant contribution of this *MLH1* founder variant in an African population. The presence of a founder variant has significant implications for genetic counselling and screening practices can also facilitate targeted surveillance strategies [55,56].

High penetrance for CRC compared to extracolonic cancers is the hallmark of LS, as observed in our study population, similar to previous studies [57,58]. The higher incidence of CRC in LSVH, as observed in this cohort, could be due to the fact that the colon is particularly susceptible to cancer because of its high turnover rate of epithelial cells, which could make it more prone to accumulating DNA replication errors leading to microsatellite instability (MSI), resulting in a higher likelihood of malignant transformation compared to other tissues [59,60,61,62]. The frequency of extracolonic cancers was relatively higher in Group 2 than in Group 1A, indicating that LSVH with germline P/LPVs in the *MSH2* MMR gene could be more susceptible to extracolonic cancers [63,64].

In the United States (U.S.), the lifetime risk of developing BC for women in the general population is approximately 12.5% (1 in 8), while the cumulative risk is ~10.9% by the age of 80 [65,66,67]. In South Africa, the lifetime risk of BC is ~1 in 27 women, which is lower compared to that reported in the U.S., while data on age specific cumulative risk are still limited [68,69,70]. In contrast, women with LS, exhibit a significantly higher lifetime and cumulative risk of BC, which varies according to the mutated MMR gene [70,71,72]. This indicates a notable increase in BC susceptibility among LSVH compared to the general population. Although the cumulative risk of BC was not determined in this study due to small number of patients with BC, it was predominantly more common in LSVH in Group 1A than in Group 2; a similar observation was made in other LS populations in previous studies [72,73,74]. A previous LS study conducted in a European population by Harkness et al. (2015) estimated the cumulative risk of breast cancer by the age of 70 years to be approximately 18.6% (95% CI 11.3 to 25.9) for LSVH with *MLH1* PV, while LSVH with *MSH2* PV was about 11.2% (95% CI 1.4 to 21.0) by the age of 70 years [72]. These findings suggest that the risk associated with breast cancer may be more pronounced in LSVH with *MLH1* PV. Our study, therefore, not only aligns with but also refines previous reports by adding a population-specific perspective, showing how these mutated MMR genes translate to higher CRC risk and certain extracolonic cancers, underscoring the need for targeted genetic counselling and surveillance strategies based on mutated MMR gene.

The disparities in the cumulative risk of any cancer and CRC between LSVH in Group 1A and Group 2 could be explained by the differing specific pathways and interactions of MMR genes in correcting DNA replication errors, as the MLH1 protein is more crucial for facilitating the repair process in conjunction with the PMS2 protein, while the MSH2 protein forms a dimer with either MSH6 or MSH3 proteins to recognize mismatches, which may contribute to the differing cancer risks observed [19,75]. Previous studies have indicated that the cumulative risk of any cancer or CRC varies between LSVH with *MLH1* and *MSH2* PV, showing that LSVH with *MSH2* PV generally exhibit a higher cumulative risk of cancer, contradicting our findings [64,76]. This could be due to the *MLH1* founder effect in our population, necessitating the need for variant-specific tailored cancer surveillance strategies in LSVH and exploring factors contributing to this variability in cancer risk. Furthermore, this observation may indicate that LSVH in Group 1 are not adhering to the recommended regular colonoscopy surveillance. Regular colonoscopies significantly reduce cancer incidence and delay the age of diagnosis by identifying and removing precancerous lesions early [77,78,79,80]. The differences in cancer risks underscore the importance of tailored surveillance strategies for individuals based on their specific mutated MMR gene [63,81]. Our findings suggest that individuals with P/PLVs in the *MLH1* gene in our African population could benefit from frequent cancer surveillance, particularly for colorectal, endometrial, and breast cancers as these were the most common cancers in this group.

The observed high cumulative risk of any cancer and CRC in males compared to females in the present study may be attributed to differences in genetic and hormonal factors [52,82]. For instance, in females, it has been reported that some tumor-suppressor genes escape the inactivation during the X-chromosome inactivation process, which may result in higher expression of these genes than in males [83,84,85]. In addition, estrogen, which is more prevalent in women, has been shown to have protective effects against certain types of cancer, such as CRC. Studies suggest that estrogen influences cellular signaling pathways that can inhibit tumor growth, leading to better survival outcomes for women compared to men with CRC [52,82,86,87,88,89,90].

Additionally, our findings indicate that the cumulative risk of CRC in male LSVH ranged from 58.6% to 91.5%, while in female LSVH, it ranged from 44.7% to 69.7%. These results are marginally higher compared to previous observations, which estimated the risk to be between 54% and 74% in male LSVH, and between 30% and 52% in female LSVH [17,91]. We also observed a higher frequency of extracolonic cancers in female LSVH (35%) in Group 1B than in male LSVH (7%), suggesting a higher incidence of extracolonic cancers in female than in male LSVH. Our observations are similar to previous studies indicating that females with LS have a significantly increased risk for various extracolonic cancers compared to males [18,92,93]. Additionally, in the same Group 1B, CRC was predominantly more common in male LSVH (93%) than in female LSVH (65%). This sex-specific disparity in our study and previous studies highlights the impact of sex on the manifestation of cancer and emphasizes the need for population-specific tailored screening and management strategies based on individual genetic profiles, including sex [17,18,19,93].

The observed significant difference in the age at CRC diagnosis between prevalent and incident cases of LSVH in Group 1B in our study population could be due to the fact that some of the individuals with the *MLH1*: c.1528C>T founder PV under our clinical surveillance were part of the clinical trial for 10 years on the daily intake of aspirin and resistant starch for the prevention of CRC [94,95,96]. This could also explain why a significant difference in age at CRC diagnosis was observed between prevalent and incident CRC cases in Group 1A but not in Group 2 (Figure 5). In addition, colonoscopic surveillance has been shown to lead to early identification of precancerous polyps and cancer, leading to the removal or early treatment of cancer, ultimately prolonging overall survival in LS, which could explain the late age at diagnosis for incident cases [77,78,79,80].

It is crucial to understand the overall survival after first cancer diagnosis in LSVH for tailored follow-up strategies to improve the overall prognosis. We found an overall 10-year crude survival rate after any cancer diagnosis of 62% in males and 78% in females, and also 70% after any LS-associated cancer, 71% after CRC, and 64% after extracolonic cancer in carriers of *MLH1*:c.1528C>T founder PV. In previous studies, the overall 10-year crude survival rate LSVH after any cancer diagnosis has been reported to be approximately 87% [12,13]. Among LSVH with *MLH1* PV, survival rates after first CRC are often favorable, with a 10-year crude survival rate of about 91%, specifically for those diagnosed with first CRC [13,24]. Our observed crude survival rates were lower compared to survival rates reported previously in other LSVH populations [12,13,16,23,24,26]. Several factors could explain this: firstly, population-specific factors such as differences in healthcare access, cancer surveillance, and early detection rates may contribute to poorer outcomes, particularly if timely intervention is less accessible, as is the case for our cohort. Secondly, genetic modifiers unique to our study population or the influence of the *MLH1*:c.1528C>T founder variant may affect survival dynamics, possibly due to variations in mutation penetrance and cancer progression. Thirdly, environmental influences, lifestyle factors, and comorbidities prevalent in our population may also impact survival outcomes, aligning with findings that disparities in lifestyle and socioeconomic factors can modulate cancer survival [97,98,99,100,101]. Our findings suggest a critical need to investigate local and genetic determinants further, which could enable the development of tailored surveillance and management protocols to improve outcomes in this population.

The main strengths of our study include:

(i) The use of the largest LS registry in Africa with genetically confirmed LSVH from different ethnic groups in South Africa.
(ii) Population-specific new insights from a unique cohort with *the MLH1*:c.1528C>T founder variant, providing valuable insights into LS in an African population.
(iii) A cumulative risk assessment using Kaplan–Meier analysis to calculate age-specific cumulative risks for cancers in LSVH by gene and sex, offering unique, personalized risk estimates specifically for an African population.
(iv) Detailed genetic characterization which offers a comprehensive breakdown of *MLH1* and *MSH2* variants, including stop codon/frameshift, missense, intronic, and deletion mutations, enhancing the understanding of the spectrum of LS variants in African population.
(v) Sex-specific cancer risk analysis which identifies significant sex-based differences in cancer risks, particularly higher CRC risk in males and extracolonic cancer risk in females, supporting tailored surveillance recommendations from an African study population.
(vi) Comparative survival analysis that examined crude survival outcomes from a new population of LSVH, comparing findings to other populations and noting lower crude survival rates, which may prompt further investigation into population-specific factors.
(vii) Clinical implications for screening that have direct implications for genetic counselling, risk prediction, and the development of customized cancer screening protocols in African LS populations.

However, our study had some limitations that can be addressed in the future studies as follows: (i) A relatively small sample size for some LS genes such as *MSH6*, *PMS2,* and *EPCAM.* (ii) We could not compare the age at diagnosis based on tumor stage due to the lack of this information in our LS registry. (iii) Expanding beyond *MLH1* and *MSH2* genes would enhance generalizability across diverse genetic backgrounds, e.g., confirmed PV in the *MSH6* and *PMS2* genes, would expand our understanding of cancer manifestation variability across all relevant MMR genes. (iv) Including more detailed surveillance and treatment data could clarify the impact of healthcare access on survival outcomes in LSVH in our study population. (v) Missing organ-specific cumulative risk of extracolonic cancers due to a low number of organ-specific cases. This could provide detailed organ-specific risk assessments in our population. (vi) We only estimated crude survival after the first cancer diagnosis for Group 1B due to a lack of survival data for Group 2 and the remainder of Group 1A. Therefore, future studies should make an effort to obtain survival information for all LSVH, which can allow for comparative survival analysis by MMR gene.

Despite the above limitations, our study provides crucial, population-specific insights into LS that fill significant knowledge gaps in the current understanding of the genetics of LS and cancer predispositions. By focusing on LSVH with variants in the *MLH1* and *MSH2* genes, as well as a unique and large cohort of LSVH with an *MLH1* founder variant in South Africa, this work brings to light gene- and sex-specific cancer risks and survival patterns previously unreported in the literature, offering invaluable data for tailoring LS management in underrepresented populations. The disparities in the cancer spectrum, risk, and survival according to MMR gene and sex, highlight the importance of taking into account the MMR gene and sex when estimating the cancer risk and survival in LSVH. These contributions are instrumental for advancing LS research and personalized care globally, underscoring the importance of including diverse populations in genetic studies to improve health equity and precision medicine.

## 5. Conclusions

Over the last three decades, South African LSVH have shown genetic and sex-based disparities influencing cancer outcomes, with *MLH1* LSVH correlated with heightened CRC risk and earlier age at cancer diagnosis than *MSH2* LSVH. Translationally, these findings underscore a groundbreaking shift toward gene- and sex-based cancer management strategies in LS, suggesting that personalized surveillance protocols will be instrumental in early detection and improved survival rates for LSVH. The implementation of these risk and survival estimates into clinical practice can significantly enhance genetic counselling and targeted prevention, especially in underrepresented African populations, advancing LS management with greater precision and equity. However, the relatively small size of our study population, i.e., only from South Africa, is a limitation, as it may affect the generalizability of our findings to other African populations. Expanding such population-specific insights globally utilizing larger cohorts holds promise for refining LS care and ensuring that risk-based cancer prevention reaches diverse communities.

## Figures and Tables

**Table 1:** Demographics and clinical characteristics of LSVH in the study cohort.

Characteristic	Group 1A, *N* = 450, n (%)	** Group 2, *N* = 120, n (%)	Group 1B, *N =* 426, n (%)	* *p*-Value
Age (years)				
Age range	14–81	21–76	14–81	NA
Sex				
Male	217 (48.2%)	40 (33.3%)	207 (48.6)	**0.005**
Female	233 (51.8%)	80 (66.7%)	219 (51.4)	
Ethnicity				
Mixed (Colored)	439 (97.5%)	108 (90.0%)	426 (100%)	**<0.001**
Indigenous African	10 (2.2%)	6 (5.0%)	0 (0.0%)	
Unknown	1 (0.2%)	6 (5.0%)	0 (0.0%)	
Cancer status				
Presymptomatic	228 (51.8%)	63 (52.5%)	222 (52.1%)	0.799
Affected	222 (49.3%)	57 (47.5%)	204 (47.9%)	
Cancer type				
CRC	176 (79.3%)	43 (75.4%)	163 (79.9%)	0.654
Extracolonic cancer	46 (20.%)	14 (24.6%)	41 (20.1%)	
Cancer by sex: Female				
CRC	68 (65.4%)	24(68.6%)	60 (64.5%)	0.890
Extracolonic cancer	36 (34.9%)	11 (31.4%)	33 (35.5%)	
Cancer by sex: Male				
CRC	108 (91.5%)	19 (86.4%)	103 (92.8)	0.715
Extracolonic cancer	10 (8.5%)	3 (13.6%)	8 (7.2%)	

Note: * *p*-value is for the comparison between Group 1A and Group 2. ** A total of seven (7) LSVH with P/LPV in the *MSH2* gene were excluded due to missing clinical and pathological data.

**Table 2:** Comparisons of mean age at cancer diagnosis between Group 1A and Group 2 stratified by cancer type and sex.

	Number of Patients (n)	Age for Group 1A in Years (n = 222)			Age for Group 2 in Years (n = 57)			Age for Group 1B in Years (n = 204)			* *p*-Value
		Mean (SD)	Median	Range	Mean (SD)	Median	Range	Mean (SD)	Median (Range)	Range	
Any first cancer											
All	279	43.2 (11.3)	41.5	16.6–78.2	47.3 (11.0)	46.4	26.0–76.0	41.7 (11.2)	42.8	15.3–78.2	**0.015**
Female	139	44.8 (11.0)	45.4	16.6–78.0	47.1 (12.0)	47.1	26.0–76.0	43,3 (10.5)	45.2	16.6–78.0	0.310
Male	140	41.8 (11.4)	40.0	15.3–78.2	47.5 (9.5)	45.8	26.6–64.9	40.4 (11.6)	40.2	15.3–78.2	**0.017**
First CRC											
All	222	41.8 (10.4)	41.5	16.6–78.2	44.8 (9.6)	44.6	26.0–64.9	41.7 (10.8)	42.8	16.6–78.2	0.078
Female	92	42.3 (9.63)	43.0	16.6–69.7	43.1 (9.3)	43.6	26.0–58.0	43.3 (10.5)	45.2	16.6–78.0	0.697
Male	127	41.6 (10.8)	39.8	19.6–78.2	46.9 (9.9)	44.6	26.6–64.9	40.4 (11.0)	39.8	19.6–78.2	**0.043**
First Extracolonic cancer											
All	60	48.3 (13.3)	49.6	15.3–78.0	54.8 (11.9)	54.6	30.0–76.0	45.8 (13.6)	49.4	15.3–78.0	0.093
Female	47	49.4 (11.9)	50.0	25.2–78.0	55.8 (13.2)	59.5	30.0–76.0	47.2 (12.2)	49.4	25.2–78.0	0.175
Male	13	44.0 (17.3)	43.8	15.3– 68.0	51.2 (5.2)	53.6	45.2–54.8	40.4 (18.8)	43.8	15.3–68.0	0.269

Note: * *p*-value is for the comparison between Group 1A and Group 2. Any first cancer includes CRC and any extracolonic cancer diagnosed in LSVH such as endometrium, bladder, brain, breast, small bowel, head and neck, liver, ovary, esophagus, pancreas, kidney, prostate, skin, spleen, gastric, thyroid, and lung.

**Table 3:** Spectrum and frequency of cancers in Group 1A, Group 2, and Group 1B.

Cancer	Group 1A (n = 222)	Group 2 (n = 57)	Group 1B (n = 204)
CRC	176 (79.3%)	43 (75.4%)	162 (79.4%)
Endometrium	15 (6.8%)	2 (3.5%)	14 (6.9%)
Bladder	1 (0.5%)	0 (0.0%)	1 (0.5%)
Brain	1 (0.5%)	0 (0.0%)	1 (0.5%)
Breast	8 (3.6%)	1 (1.8%)	6 (2.9%)
Small bowel	9 (4.1%)	3 (5.3%)	8 (3.9%)
Head and Neck	0 (0.0%)	2 (3.5%)	0 (0.0%)
Liver	1 (0.5%)	0 (0.0%)	1 (0.5%)
Ovary	2 (0.9%)	0 (0.0%)	2 (0.9%)
Esophagus	0 (0.0%)	1 (1.8%)	0 (0.0%)
Pancreas	1 (0.5%)	0 (0.0%)	1 (0.5%)
kidney	1 (0.5%)	0 (0.0%)	1 (0.5%)
Prostate	1 (0.5%)	0 (0.0%)	1 (0.5%)
Skin	2 (0.9%)	1 (1.8%)	2 (0.9%)
Spleen	0 (0.0%)	1 (1.8%)	0 (0.0%)
Gastric	3 (1.4%)	2 (3.5%)	2 (0.9%)
Thyroid	1 (0.5%)	0 (0.0%)	1 (0.5%)
Lung	0 (0.0%)	1 (1.8%)	0 (0.0%)

**Table 4:** Cumulative risk (%) of any cancer, colorectal, and extracolonic cancers diagnosis by age, sex, and gene in LSVH.

Cancer Type	Age (Year)	All % (CI95)	Sex		Group 1A			Group 2			Group 1B		
			Female % (CI95)	Male % (CI95)	All % (CI95)	Female % (CI95)	Male % (CI95)	All % (CI95)	Female % (CI95)	Male % (CI95)	All % (CI95)	Female % (CI95)	Male % (CI95)
Any cancer	50	50.4 (45.0–55.3)	44.2 (36.8–50.7)	58.0 (49.6–65.0)	54.2 (47.9–59.7)	48.0 (39.1–55.6)	60.9 (51.6–68.4)	37.7 (26.3–47.2)	34.0 (22.8–60.6)	44.8 (22.8–60.6)	54.9 (48.3–60.6)	49.3 (42.8–57.2)	60.8 (53.1–69.1)
	60	72.7 (66.9–77.5)	66.8 (58.3–73.5)	80.0 (71.4–86.1)	74.8 (68.1–80.2)	69.7 (59.5–77.4)	80.3 (70.6–86.8)	65.5 (51.8–75.3)	59.0 (41.50–71.30)	78.4 (51.4–90.4)	73.4 (66.4–79.0)	66.9 (62.5–74.9)	80.0 (69.2–86.8)
	70	87.1 (80.8–91.3)	81.0 (70.9–87.6)	93.5 (85.0–97.2)	88.4 (81.2–92.8)	82.3 (70.3–89.4)	94.1 (84.2–97.8)	82.9 (65.2–91.6)	77.7 (51.9–89.7)	91.9 (55.6–98.5)	87.0 (79.4–91.8)	78.5 (61.4–86.0)	94.0 (83.9–97.8)
Colorectal	50	51.5 (45.5–56.9)	44.7 (36.2–52.1)	58.6 (49.8–65.8)	54.5 (47.6–60.5)	47.1 (36.7–55.7)	61.3 (51.5–69.1)	41.3 (28.3–51.9)	38.4 (22.2–51.3)	46.1 (22.3–62.6)	54.6 (47.3–60.8)	47.5 (43.5–56.5)	60.9 (51.0–69.0)
	60	70.3 (63.5–75.8)	60.8 (50.6–68.8)	79.3 (70.0–85.8)	71.7 (63.9–77.8)	60.7 (48.6–70.0)	80.4 (70.0–87.1)	65.2 (49.4–76.1)	59.7 (38.8–73.5)	74.6 (44.5–88.4)	71.2 (63.1–77.5)	58.7 (47.8–68.3)	80.6 (70.2–87.5)
	70	82.1 (73.7–87.7)	69.7 (54.5–79.8)	91.5 (82.1–96.0)	84.1 (74.6–90.0)	71.9 (53.9–82.9)	92.1 (81.1–96.7)	75.0 (53.8–86.4)	59.7 (38.8–73.5)	90.5 (48.4–98.2)	82.3 (72.9–88.4)	65.1 (53.0–75.4)	92.2 (81.3–96.7)
Extracolonic	50	15.2 (9.4–20.6)	16.5 (9.3–23.1)	11.4 (2.3–19.7)	18.5 (11.0–25.4)	20.9 (11.4–29.4)	11.2 (1.4–19.9)	5.8 (0.0–12.0)	4.6 (1.1–10.6)	11.1 (0.0–29.4)	19.5 (11.4–27.8)	22.2 (12.1–31.2)	10.6 (0.2–19.9)
	60	38.1 (27.5–47.1)	39.5 (27.3–49.6)	34.0 (14.8–51.8)	42.1 (28.8–53.0)	46.5 (30.7–58.7)	28.3 (0.9–48.0)	27.7 (9.4–42.4)	23.1 (6.9–38.5)	46.7 (24.9–74.9)	36.9 (25.8–48.5)	41.3 (26.2–53.3)	21.8 (0.0–41.1)
	70	59.8 (29.0–70.7)	59.8 (43.3–71.7)	60.4 (31.5–81.9)	61.3 (42.3–74.1)	61.1 (41.0–74.4)	64.1 (12.8–100.0)	56.2 (20.7–75.8)	58.2 (14.2–79.6)	46.7 (24.9–74.9)	57.9 (37.8–74.6)	57.3 (36.4–71.4)	60.9 (0.0–85.6)

Note: Any first cancer includes CRC and any extracolonic cancer diagnosed in LSVH such as endometrium, bladder, brain, breast, small bowel, head and neck, liver, ovary, esophagus, pancreas, kidney, prostate, skin, spleen, gastric, thyroid, and lung.

**Table 5:** Crude survival after first cancer diagnosed before the age of 65 years by sex and cancer type in Group 1B.

	Number of LSVH	Number of Deaths	5-Year Survival (95% CI)	10-Year Survival (95% CI)
Male	107	43	73.0% (64.9–82.7%)	62.1% (52.9–74.1%)
Female	90	26	81.1% (73.1–90.0%)	77.6% (68.8–87.5%)
Any cancer	197	69	76.9% (70.9–83.3%)	69.5% (62.6–77.1%)
CRC	161	56	78.5% (72.1–85.4%)	70.9% (63.5–79.2%)
Any extracolonic cancer	36	13	70.0% (55.8–87.7%)	63.6% (47.4–85.3%)
Gynaecological cancer	16	5	86.5% (72.1–85.4%)	74.2% (70.7–100%)
Diagnosed < 40 years	90	27	81.4% (73.6–90.1%)	73.3% (64.0–84.1%)
Diagnosed > 40 years	107	42	72.6% (64.0–82.4%)	65.9% (56.3–77.2%)

Note: Any first cancer includes CRC and any extracolonic cancer diagnosed in LSVH such as endometrium, bladder, brain, breast, small bowel, head and neck, liver, ovary, esophagus, pancreas, kidney, prostate, skin, spleen, gastric, thyroid, and lung.

## Data Availability

The data that support the findings of this study are available from the corresponding author upon reasonable request. The data are not publicly available due to [restrictions requiring prior approval for sharing raw data].

## Supplementary Materials

The following supporting information can be downloaded at: https://www.mdpi.com/article/10.3390/biomedicines12122906/s1, Figure S1: The distribution of age at any first cancer diagnosis in *MLH1* and *MSH2* pathogenic variant carriers; Figure S2: Crude survival after first cancer diagnosis by sex and tumor location; Table S1: Summary of mean age at diagnosis for major cancers diagnosed in LSVH.
