# Supplementary material for: Genomic Medicine in the Developing World: Cancer Spectrum, Cumulative Risk and Survival Outcomes for Lynch Syndrome Variant Heterozygotes with Germline Pathogenic Variants in the MLH1 and MSH2 Genes
Source: Biomedicines. 2024 Dec 20;12(12):2906. doi: 10.3390/biomedicines12122906 (PMC11672899; doi:10.3390/biomedicines12122906)
Supplement: Supplementary file 1 [file biomedicines-12-02906-s001.zip › Supplementary Figure S1.pdf]

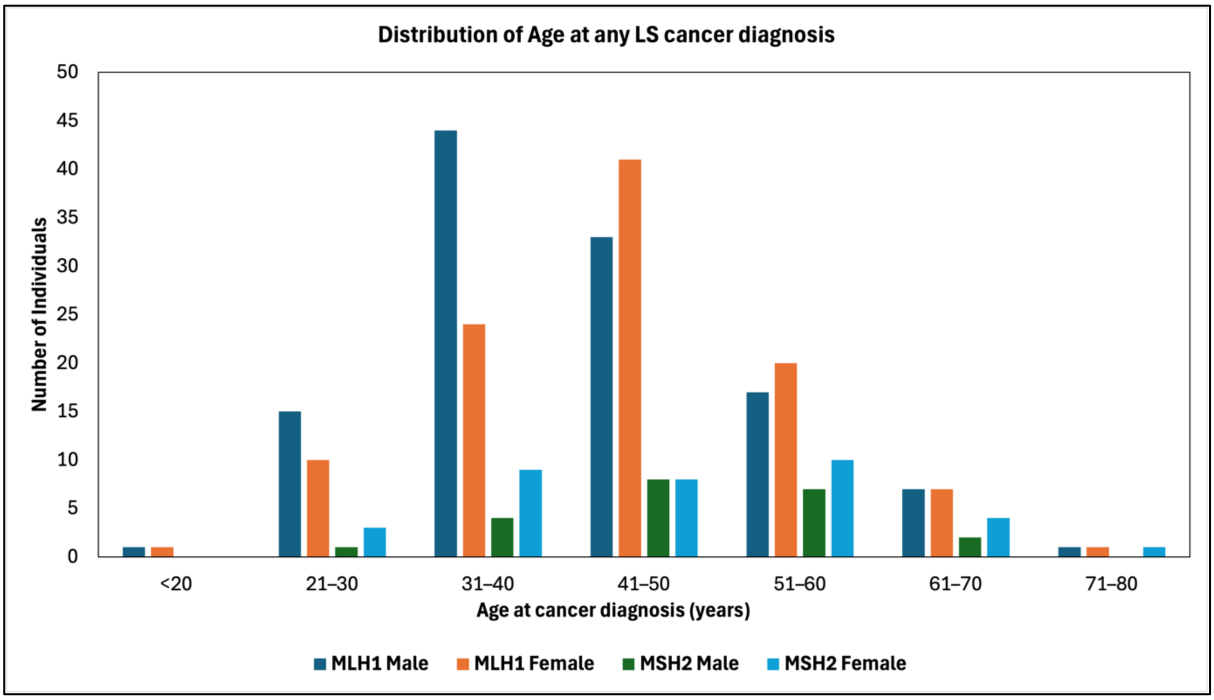

Supplementary Figure S1: The distribution of age at any first cancer diagnosis in *MLH1* and *MSH2* pathogenic variant carriers. This figure illustrates that males with P/LV in the *MLH1* gene were mostly diagnosed with cancer in the age group of 31-40 years, while females were mostly diagnosed in the age group of 41-50 years.
