# Supplementary material for: Genomic Medicine in the Developing World: Cancer Spectrum, Cumulative Risk and Survival Outcomes for Lynch Syndrome Variant Heterozygotes with Germline Pathogenic Variants in the MLH1 and MSH2 Genes
Source: Biomedicines. 2024 Dec 20;12(12):2906. doi: 10.3390/biomedicines12122906 (PMC11672899; doi:10.3390/biomedicines12122906)
Supplement: Supplementary file 1 [file biomedicines-12-02906-s001.zip › Supplementary Figure S2.pdf]

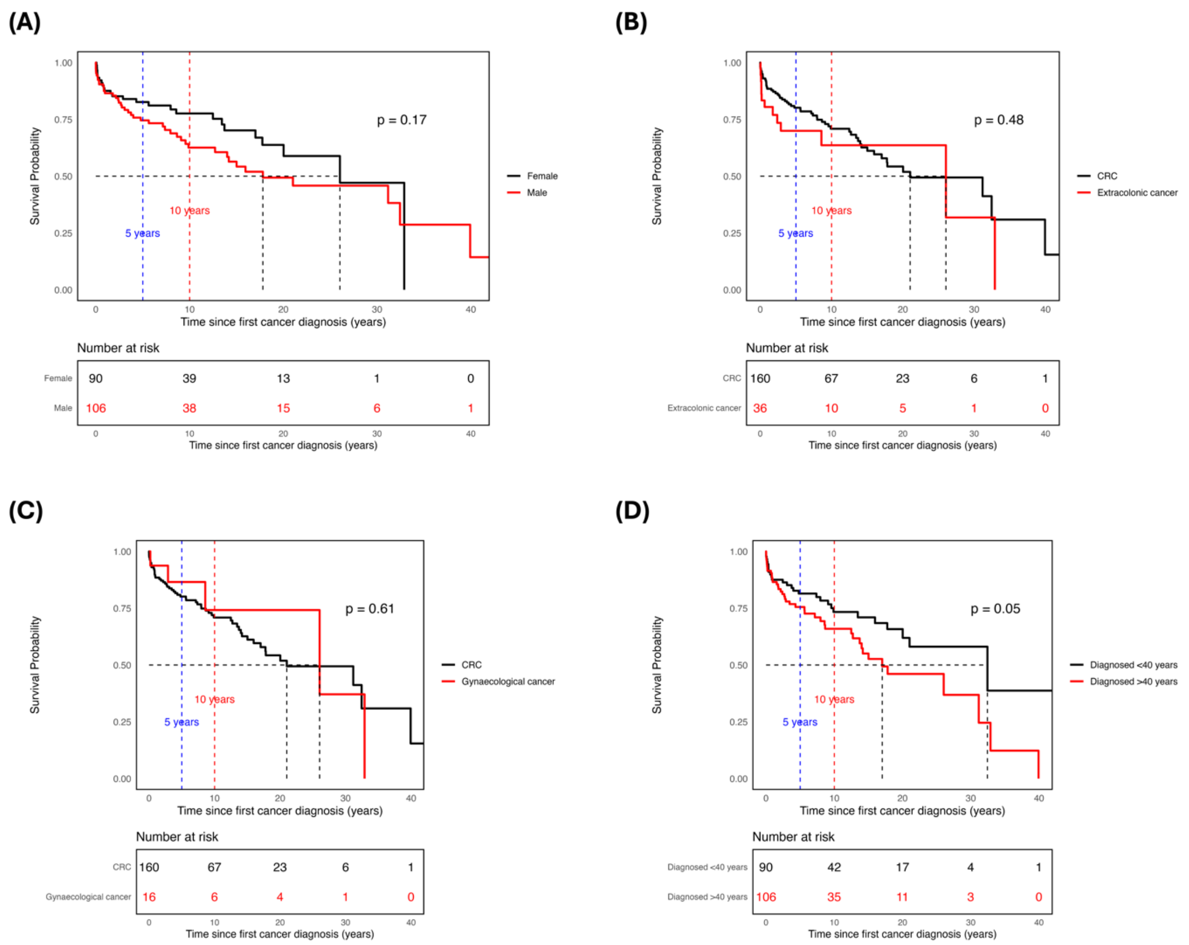

Supplementary Figure S2: Crude survival after first cancer diagnosis by sex and tumor location. (**A**) shows the crude survival after any first LS cancer diagnosis stratified by sex (female = black and male = red), with the dotted lines showing 5-year (blue) and 10-year (red) survival. (**B**) shows the crude survival after the first CRC (black) and extracolonic cancer (red) diagnosis. (**C**) shows the crude survival after the first CRC (black) and gynaecological cancer (red) diagnosis. (**D**) shows the crude survival after any first cancer diagnosed below the age of 40 (black) and above the age of 40 (red) years. Then median survival age after the first cancer diagnosis is indicated by a dotted black line.
