# Supplementary material for: Genomic Medicine in the Developing World: Cancer Spectrum, Cumulative Risk and Survival Outcomes for Lynch Syndrome Variant Heterozygotes with Germline Pathogenic Variants in the MLH1 and MSH2 Genes
Source: Biomedicines. 2024 Dec 20;12(12):2906. doi: 10.3390/biomedicines12122906 (PMC11672899; doi:10.3390/biomedicines12122906)
Supplement: Supplementary file 1 [file biomedicines-12-02906-s001.zip › Supplementary Table S1.pdf]

Supplementary Table S1: Summary of mean age at diagnosis for major cancers diagnosed in LSVH.

Cancer	n	Mean age at diagnosis (years)	Age Range (years)
CRC	217	41.2	17–78
Endometrium	17	49.7	32–63
Breast	9	53.8	34–70
Small bowel	12	41.2	15–67
Skin	3	65.0	54–78
Gastric	5	49.0	32–62
